# Complete denture tooth arrangement technology driven by a reconfigurable rule

**DOI:** 10.1371/journal.pone.0198252

**Published:** 2018-06-18

**Authors:** Ning Dai, Xiaoling Yu, Qilei Fan, Fulai Yuan, Lele Liu, Yuchun Sun

**Affiliations:** 1 College of Mechanical & Electrical Engineering, Nanjing University of Aeronautics &Astronautics, Nanjing, P.R.China; 2 Center of Digital Dentistry, Faculty of Prosthodontics, Peking University School and Hospital of Stomatology, Beijing, P.R.China; School of Dentistry of the University of Sao Paulo, BRAZIL

## Abstract

The conventional technique for the fabrication of complete dentures is complex, with a long fabrication process and difficult-to-control restoration quality. In recent years, digital complete denture design has become a research focus. Digital complete denture tooth arrangement is a challenging issue that is difficult to efficiently implement under the constraints of complex tooth arrangement rules and the patient’s individualized functional aesthetics. The present study proposes a complete denture automatic tooth arrangement method driven by a reconfigurable rule; it uses four typical operators, including a position operator, a scaling operator, a posture operator, and a contact operator, to establish the constraint mapping association between the teeth and the constraint set of the individual patient. By using the process reorganization of different constraint operators, this method can flexibly implement different clinical tooth arrangement rules. When combined with a virtual occlusion algorithm based on progressive iterative Laplacian deformation, the proposed method can achieve automatic and individual tooth arrangement. Finally, the experimental results verify that the proposed method is flexible and efficient.

## Introduction

Dentition loss is a common, frequently occurring disease of the elderly, and complete dentures are the most important conventional treatment method for this disease. Complete denture restoration designs need to meet specific mechanical, biological and aesthetic requirements. The traditional hand-made design and fabrication process for complete dentures is complex and involves multiple processes. Furthermore, the quality is difficult to control.

CAD/CAM (computer-aided design/computer-aided manufacturing) technology has been widely used in the dental field, and its use is promoting digital complete denture technology in the direction of high efficiency, high precision and automation. The increasing application of dental scanning equipment in dental clinics [1,2] and the use of ceramic denture three-dimensional printing [3,4], as well as resin-based high-precision multi-axis manufacturing technology [5,6], have provided a basis for the digital fabrication of complete dentures. However, the design of complete dentures is still a technical bottleneck due to the complex fabricating processes.

The computer-aided arrangement of artificial teeth is one of the key technologies in the design of complete dentures. The literature can be divided into three categories: (1) Digitizing the manual pre-arrangement of the teeth. Maeda et al. [7] produced a complete denture after the registration of a manually pre-arranged artificial teeth data with the baseplate data. Goodacre et al. [8] developed the software to realize virtual assembly with the baseplate data, jaw records and pre-arranged artificial teeth. Although this method is easy to carry out, it does not achieve complete virtual tooth arrangement. (2) Robot-assisted tooth arrangement. Lü et al. [9] conducted the positioning and measurement of an edentulous plaster model and used the power function to generate a tooth arrangement curve that drove the robot arm to generate an automatic arrangement of artificial teeth. Zhang et al. [10] designed a tooth arrangement mechanical arm for automatic tooth arrangement of a complete denture. This type of technology has a complex operation process, and its efficiency is not high. (3) Modeling of the artificial tooth arrangement process. Busch et al. [11] studied the anatomic shape of the jaw and proposed a method that generates a structure on the alveolar crest of the edentulous maxilla and mandible. This structure intersects with the occlusal plane to obtain the dental arch curve. The dental arch curve was used as a reference for the arrangement of artificial teeth. Sun et al. [12] proposed a method that uses the quantitative constraints of traditional complete denture fabrication to achieve computer-aided tooth arrangement, but the flexibility and adaptability of the tooth arrangement process still need to be improved. Baba [13] analyzed and evaluated the four existing commercial complete denture digital fabrication systems and found that the balanced occlusion of digital tooth arrangements still needs further improvement. Ahmat et al. [14] proposed the use of industrial CAD software to model the digital tooth arrangement of a complete denture, but the modeling required a great deal of manual interactive operations and had a low degree of automation. The medical rules for the artificial tooth arrangement of complete dentures are complex, and tooth arrangement theory is still being developed; currently, there are no reports of an automatic tooth arrangement method for complete dentures.

The present paper proposes a rule-driven, automated complete denture tooth arrangement method based on a dynamic and reconfigurable process and establishes an artificial tooth characteristics database that is associated with individualized constraint features. The dynamic tooth arrangement rule of the tooth arrangement module drives rapid automatic tooth arrangement, thereby providing a useful method for the digital design of complete dentures.

## Materials and methods

### Technology flowchart

**Fig 1:**
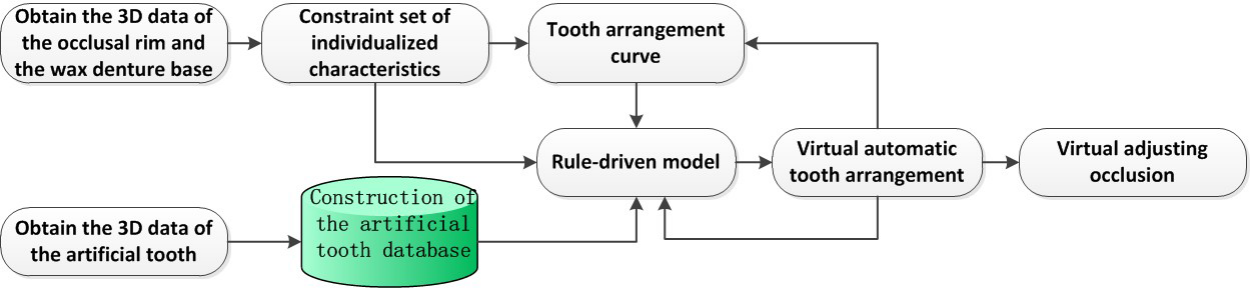
Technology flowchart of automatic tooth arrangement for complete dentures.

The automated tooth arrangement process for complete dentures includes the following parts as shown in Fig 1. ① Obtaining the patient’s three-dimensional data: Three-dimensional scanning of a plaster model of the patient’s teeth and of the premade wax occlusal rim is performed to obtain the three-dimensional data of the edentulous surface. ② Obtaining the constraint set of individualized characteristics: Extraction of the patient’s individualized characteristic constraint sets, such as the maxillary and mandibular alveolar crest curves and the high lip line, is performed based on the edentulous model and the wax occlusal rim to generate the maxillary and mandibular anterior and posterior tooth arrangement curves. ③ Construction of an artificial tooth characteristics database: Standard artificial teeth are scanned, and the characteristic constraint point set and the coordinate system of the artificial tooth are defined. ④ Establishment of a rule-driven model: A model that is based on the medical tooth arrangement principle and includes the characteristic constraints and the contact constraints is established to drive the automated virtual tooth arrangement of the artificial tooth. ⑤ Virtual occlusion: Virtual occlusion is performed for the maxillary and mandibular occlusal surfaces to achieve complete denture tooth arrangement.

### Obtaining the individualized characteristics constraint set

**Fig 2:**
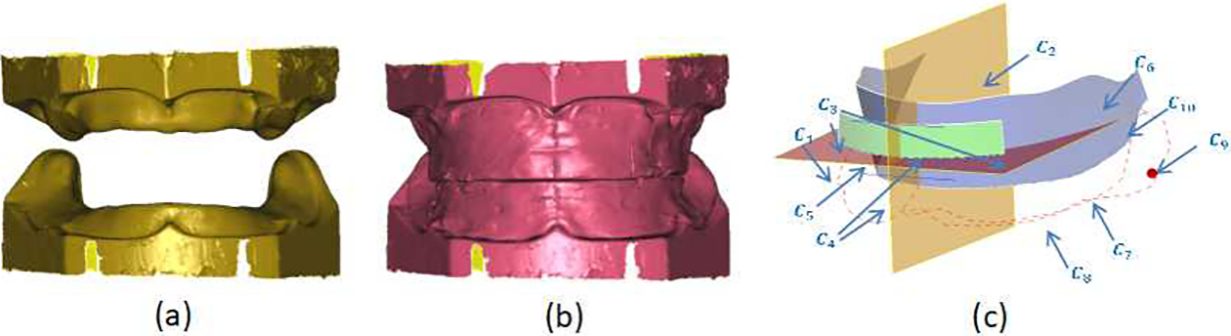
Obtaining the individualized characteristics constraint set. (a) Edentulous digital model; (b) premade occlusal rim digital model; (c) extracted individualized characteristics constraint set.

Scanning is used to obtain the edentulous maxillary and mandibular model and the three- dimensional digital model of the wax occlusal rim, as shown in Fig 2(A) and 2(B). The patient’s edentulous maxilla and mandible and the premade wax occlusal rim include the patient’s individualized anatomical features; these are obtained one by one by defining the curves and surfaces and are stored structurally using XML files, as shown in Fig 2(C). The defined individualized characteristics constitute the constraint information set {*C*_*i*_}, as shown in Table 1.

**Table 1:** Individualized characteristics constraint information.

Constraint type	Constraint name	Method of obtaining the constraint
Aesthetic constraints	*C*_1_: Occlusal plane	Fit maxillary and mandibular rim contact surfaces
	*C*_2_: Median sagittal plane	Symmetrical vertical occlusal plane
	*C*_3_: Left and right mouth lines	Direct extraction from digital occlusal rim
	*C*_4_: High and low lip line	Direct extraction from digital occlusal rim
	*C*_5_: Fullness constraint surface	High lip line and left and right mouth lines intersect the wax occlusal rim surface
Anatomical constraints	*C*_6_: Maxillary alveolar crest line	Interactive extraction from edentulous maxillary model
	*C*_7_: Mandibular alveolar crest line	Interactive extraction from edentulous mandibular models
	*C*_8_: Base edge line	Interactive extraction from edentulous maxillary and mandibular models
	*C*_9_: Mandibular left and right molar rear pad points	Interactive extraction from edentulous mandibular models
Association constraint	*C*_10_: The upper and lower alveolar crest formed surface	Interpolation from the maxillary and mandibular alveolar crest curves

### Construction of an artificial tooth database

**Fig 3:**
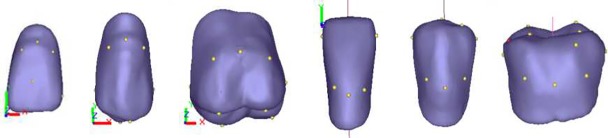
Defining artificial tooth characteristics. (a) Maxillary left 1st incisor; (b) maxillary left 4th premolar; (c) maxillary left 6th molar; (d) mandibular left 1st incisor; (e) mandibular left 4th premolar; and (f) mandibular left 6th molar.

The brand of artificial teeth is the Heraeus.To support the follow-up tooth arrangement rule, it is necessary to construct a coordinate system for the artificial tooth and to define its characteristics. Because different types of teeth display large differences in morphology and function and the positioning characteristics of teeth during arrangement also have differences, it is necessary to define the characteristics according to the function. The local coordinate system is used to control the positional relationship between the occlusal surface and the occlusal plane, the fullness mark points and the long axis of the tooth are used to control tooth posture, the central fossa is used to restrain the buccolingual position of the posterior teeth, and the cusp and the mid-buccal groove mark are used to determine the staggered relationship of the cusp and socket of the posterior tooth, as shown in Fig 3. "+" stands for “needs," and "-" stands for "does not need." The construction characteristics of a standard tooth are shown in Table 2. Taking the left artificial teeth as an example, the numbering rules are illustrated. The right side artificial teeth are in the same way, but the subscript is changed to "R".

**Table 2:** Characteristic information for artificial teeth.

Tooth type	Local coordinate system	Fullness mark point	Tooth long axis	Central fossa	Cusp	Mid-buccal groove mark points		Mesiodistal contact points
Maxillary anterior teeth $T_{Li}^{T},i=1,2$	+	+	-	-	-		-	+
Maxillary premolar $T_{L4}^{T}$	+	-	+	-	+		-	+
Maxillary molar $T_{Li}^{T},i=5,6,7$	+	-	-	-	+		-	+
Mandibular anterior teeth $T_{Li}^{D},i=1,2$	+	-	+	-	-		-	+
Mandibular premolars $T_{L4}^{D}$	-	-	+	+	+		-	+
Mandibular molars $T_{Li}^{D},i=5,6,7$	-	-	+	+	+		+	+

Note: In the formula, T sup “T” means “Top”, “D” means “Down”. “T” represents the maxillary teeth, and the “D” represents the mandibular teeth. T sub “L” means “Left”, “R” means “Right”. “L” represents the left tooth, and “R” represents the right tooth.

### Rule-driven virtual tooth arrangement framework

The traditional artificial tooth arrangement method provides the theoretical basis for the computer-aided tooth arrangement; however, because there is no generalized quantitative description and the actual situation in the patient’s mouth is complex, there is still no generalized streamlined tooth arrangement method. Based on the fact that there is a certain constraint relationship between the position of the full dentition and the individualized characteristics of the edentulous jaw, the present paper proposes a constraint rule-driven virtual tooth arrangement frame with a dynamic and reconfigurable process.

#### Geometric constraint operator

Position constraint operator *P*: Position constraints can allow standard dentition to have a preliminary arrangement according to functional regions, and defining the tooth arrangement curve can guide the arrangement of dentition in each functional region. The tooth arrangement curve of the maxillary anterior teeth *P*_1_ = *C*_1_ ∩ *C*_5_, is defined by the intersection of the occlusal plane *C*_1_ and the fullness constraint surface *C*_5_, which guides the edges of the maxillary anterior teeth to arrange along the tooth arrangement curve. The tooth arrangement curve of the mandibular anterior tooth *P*_2 =_ *Offset*(*P*_1_,*d*_0_) is formed by *P*_1_ in the occlusal plane with an offset along the lingual direction, and the offset distance *d*_0_ = 1~3*mm*, which guides the mandibular anterior tooth edge to arrange along the tooth arrangement curve. The posterior tooth arrangement curve (to distinguish the tooth arrangement order of the posterior tooth, the maxillary and mandibular posterior tooth arrangement curves are defined as *P*_4_ and *P*_5_, respectively) is defined by the straight line from the mandibular 2nd lateral incisor far middle contact points to the molar rear pad characteristic point *C*_9_, which guides the posterior central fossa to arrange in order along the posterior tooth arrangement curve. The position restraint *P*_3_ of the canine is determined by the contact relationship of the initially arranged adjacent teeth.

Proportion constraint operator *S*: The actual size of the dental arch and the smiling curve position of the patient are not inconsistent. Therefore, it is necessary to perform an individualized adjustment of the size of the standard tooth. By collecting the left and right mouth line *C*_3_ and the high and low lip line *C*_4_, we can calculate the mesiodistal proportion coefficient *α*_1_ for the size constraint *S*_1_ of the maxillary anterior teeth, the cervico-occlusal proportion coefficient *β*_1_, the mesiodistal proportion coefficient α_2_ for the size constraint *S*_2_ of the mandibular anterior teeth, and the cervico-occlusal proportion coefficient *β*_2_, where *D*(∙) stands for the mesiodistal distance of the current tooth, and *L*_*TOP*_ and *L*_*down*_ represent the arc lengths of the maxillary anterior tooth arrangement curve and the mandibular anterior tooth arrangement curve, respectively. *H*_*up*_ and *H*_*down*_ represent the distance from the high lip line and the low lip line to the occlusal plane *C*_1_, respectively, and *ε*_0_,*ε*_1_,*ε*_2_ and *ε*_3_ are the correction values, which the present paper takes as 0, 0, 1.5 and 1.7, respectively.

$$\alpha_{1}∙\sum_{i=1}^{i\leq 3}\left(D(T_{Li}^{T})+D(T_{Ri}^{T})\right)=L_{Top}+\varepsilon_{0}$$ (1)

$$\alpha_{2}∙\sum_{i=1}^{i\leq 3}\left(D(T_{Li}^{D})+D(T_{Ri}^{D})\right)=L_{down}-{D(T}_{L3}^{T})+\varepsilon_{1}$$ (2)

$$\beta_{1}{∙L}_{LUi}=H_{up}∙\varepsilon_{2}$$ (3)

$$\beta_{2}∙L_{LDi}=H_{down}∙\varepsilon_{3}$$ (4)

The proportion coefficient *γ* of the posterior tooth size constraint *S*_3_ is defined by the distance between the far midpoint of the maxillary anterior tooth and the characteristic point of the molar rear pad, where *L*_*back*_ represents the length of the posterior tooth arrangement curve, and *ε*_4_ is the correction value; in general, *ε*_4_ = 0.

$$\gamma ∙\sum_{i=4}^{i\leq 7}D(T_{Li}^{T})=L_{back}+\varepsilon_{4}$$ (5)

**Fig 4:**
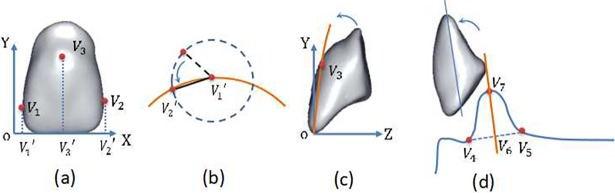
Posture adjustment of anterior teeth. (a) Defining the maxillary central incisor characteristics; (b) posture rotation of the maxillary central incisor on the tooth arrangement curve; (c) the fullness mark of the maxillary central incisor rotates to the fullness constraint surface; and (d) adjusting the long axis posture of the mandibular central incisor.

**Fig 5:**
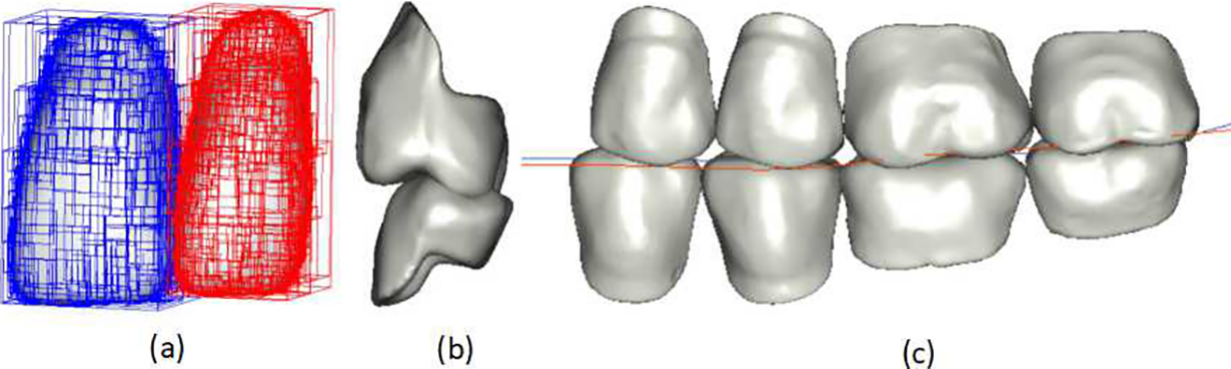
Contact constraint model. (a) Adjacent teeth positioned by Loose Octree; (b) occlusal contact of the first maxillary and mandibular molars; (c) occlusal contact of the posterior dentition.

Posture constraint operator *R*: Posture constraint is used to define the spatial posture of an artificial tooth on the characteristic constraint curve (such as the anterior tooth arrangement curve or the posterior tooth arrangement curve). When arranging the maxillary anterior teeth, the maxillary anterior tooth arrangement curve and the fullness constraint surface are used as the targets to constrain and define the maxillary anterior tooth posture. First, the maxillary central incisor is inserted, and the $V_{1}^{\prime }$ point is moved to the intersection of the maxillary anterior tooth arrangement curve and the median sagittal plane (*V*_1_ and *V*_2_ are the points of teeth, and their projection points on the local coordinate axis X are $V_{1}^{\prime }$ and $V_{2}^{\prime }$); then, using the mesiodistal distance as the radius, the $V_{2}^{\prime }$ point is moved to the maxillary anterior tooth arrangement curve. Finally, the fullness mark *V*_3_ is rotated along the $V_{1}^{\prime }V_{2}^{\prime }$ axis to the fullness constraint surface *C*_5_, as shown in Fig 4(A)–4(C). The mandibular anterior tooth posture control does not have a fullness surface constraint. In that case, we first calculate the midpoint *V*_6_ of the base boundary points *V*_4_ and *V*_5_ and the vector $\vec{V_{6}V_{7}}$ of the alveolar crest *V*_7_, adjusting the long axis of mandibular anterior tooth to be parallel to $\vec{V_{6}V_{7}}$ to constrain the posture of the mandibular anterior teeth in Fig 4(D). The procedure for the posture constraint of the posterior teeth can be divided into two steps. Step one is the preliminary posture constraint, which directly defines the posterior tooth posture and the positional relationship to the coordinate system planes xoz in the coordinate system of the artificial tooth database; the coincidence of the coordinate system planes with the occlusal plane is used to determine the initial posterior tooth posture. Step two is the overall posture constraint of the posterior tooth dentition. Here, the buccal tip of the dentition of the initially positioned maxillary posterior teeth forms the longitudinal occlusal curve, as shown by the orange curve in Fig 5(C); the corresponding mandibular posterior buccal tip corresponds to the maxillary posterior central fossa starting from the mandibular 6^th^ molar for the rearrangement.

#### Contact constraint operator *F*

Adjacent tooth contact constraint *F*_1_: During the arrangement of artificial teeth, it is necessary to maintain contact of the teeth in real time, so the adjacent teeth require 0–0.01 mm interference to fully contact. The use of a hierarchical bounding box can speed up real-time collision detection. Considering the effect of tooth scaling on the hierarchical bounding box, we use Loose Octree to perform space division for adjacent teeth; then, using the Separating Axis Theorem (SAT) [15] to detect the collision, we take the average distance of the points in the interference region as the amount of interference, as shown in Fig 5(A).

Occlusal contact constraint *F*_2_: The occlusal contact relationship is a key factor in recovering correct chewing movement. According to the correct occlusal relationship between the maxillary and mandibular posterior teeth, the occlusal contact constraint is defined. First, the central fossa of the initially positioned first mandibular molar is arranged along the tip curve of the maxillary posterior teeth; second, the mandibular first molar is rotated along the central fossa to the occlusal contact position, as shown in Fig 5(B). Finally, the adjacent tooth achieves occlusal contact constraint in order, as shown in Fig 5(C).

#### Reconfigurable rule *R* drives tooth arrangement

The strategy for complete denture tooth arrangement is not unique. Based on the above constraint models, various rule-driven typical tooth arrangement processes will be implemented according to the conditions of different patients. In the virtual tooth arrangement frame shown in Fig 4, the sequential rule R_1_ = {S_1_,P_1_,R_1_,P_2_,R_2_,P_3_,R_3_,P_5_…}, representing ① S_1_: the maxillary central incisor $T_{L1}^{T}$ and the lateral incisor $T_{L2}^{T}$ achieve scaling under the {C_3_,C_4_,C_9_} characteristics restraint; ② P_1_: $T_{L1}^{T}$ and $T_{L2}^{T}$ are positioned on the maxillary anterior tooth arrangement curve under {C_1_,C_2_,C_6_,C_7_,C_8_,C_9_,C_10_} characteristics constraint; ③ R_3_: $T_{L1}^{T}$ and $T_{L2}^{T}$ have posture control under {C_1_,C_2_,C_5_,C_6_,C_7_} characteristics constraint; ④ P_2_: the mandibular central incisor $T_{L1}^{D}$ and the lateral incisor $T_{L2}^{D}$ are positioned on the mandibular anterior tooth arrangement curve under the characteristics constraint; ⑤ R_2_: $T_{L1}^{D}$ and $T_{L2}^{D}$ have posture control under the characteristics constraint; ⑥ P_3_,R_3_ are first used to position the maxillary canines, followed by posture adjustment; and ⑦ P_5_ is used to achieve the initial arrangement of the mandibular dentition. According to the different tooth arrangement strategies, the tooth arrangement rules can be dynamically reconfigured.

**Fig 6:**
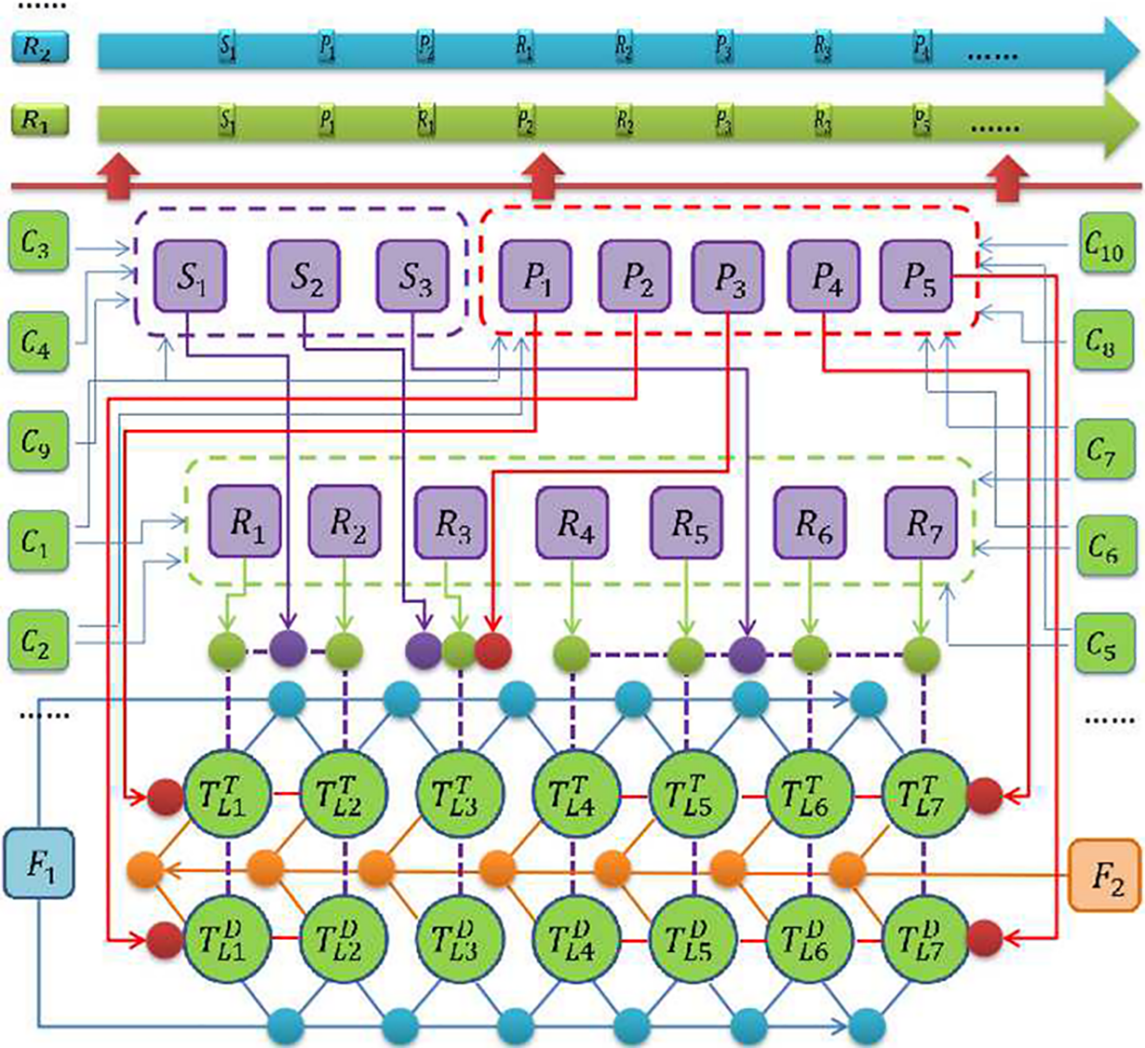
Tooth arrangement frame based on the rules.

By establishing a characteristic constraint operator, the tooth arrangement process is reconfigured according to tooth arrangement rules. According to the above constraints, all artificial teeth are arranged in sequence, as shown in Fig 6.

### Virtual adjusting occlusion

**Fig 7:**
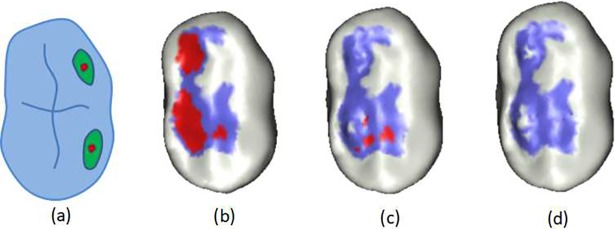
Virtual adjusting occlusion. (a) Occlusal region analysis; (b) occlusal interference region detection: (c) results after 10 iterative processes; (d) results after 20 iterative processes. Interference distances greater than 0.05 mm are shown in red, and interference distances less than 0.05 mm are shown in light blue.

To form a good occlusal relationship between opposing teeth, it is necessary to adjust the occlusal surfaces of the teeth to eliminate or reduce adjustment and grinding when the patient wears the teeth. Considering that Laplacian coordinates [16] can describe the local geometric details of a surface and can maintain the anatomical characteristics of the occlusal plane during a deformation process, we proposed the use of progressive iterative Laplacian deformation to achieve virtual adjusting occlusion. First, the method presented in Section 2.4.2 is used to calculate the interference region as the area to be deformed; the point set in the region {*v*_*i*_,*i* = 1,…,*n*} is shown as the green area in Fig 7(A). The Laplacian coordinates {*v*_*i*_} Δ = {*δ*_*i*_} are

$$\delta_{i}=\sum_{j\in N(i)}\omega_{ij}(v_{i}-v_{j})$$ (6)

*N*(*i*) is the set of 1-ring neighborhood points adjacent to *v*_*i*_, *ω*_*ij*_ represents the weight of the connection of the vertices *v*_*i*_ and *v*_*j*_, and ∑_*j*∈*N*(*i*)_*ω*_*ij*_ = 1 (usually *ω*_*ij*_ = 1/*d*_*i*_, and *d*_*i*_ is the number of points that are next to *v*_*i*_.). The matrix represents Laplacian coordinate Δ = LV, where L is the Laplacian operator for n × n dimensions. Second, we select the point set with a larger distance in the interference region as the handle point set, as shown in the red area in Fig 7(A). Each time the handle point set is moved 0.5 * *D*_*max*_ distance toward the opposing jaw along the average normal vector direction of the region, the point position in the deformed region is updated; this iterative approximation is repeated until it meets the interference requirement of the occlusal surface. To reduce the distortion phenomenon in the deformed region, the minimizing equation for *E*(*V*′) is used to calculate the point displacement in the deformed region [17].

$$E(V^{\prime })=\sum_{i=1}^{n}{\left\|\delta_{i}-\delta_{i}^{\prime }\right\|}^{2}+\sum_{i=m}^{n}{\left\|v_{i}^{\prime }-u_{i}\right\|}^{2}$$ (7)

$\delta_{i}^{\prime }$ is the Laplacian coordinate of the target vertex, and $v_{i}^{\prime }$ is the handle point position after the deformation, and *u*_*i*_ is the target position of the handle point.

The above problems are converted into optimization problems with location constraints

$${AV}^{‘}=b$$

$A=\left[\begin{array}{c} L\\ H \end{array}\right]$, $b=\left[\begin{array}{c} \Delta \\ h \end{array}\right]$, H is an *m* × *n*-dimensional matrix representing the weight of the constraint point, and *h* = {*h*_*i*_,*i* = *n* – *m*} is the *m* × 3-dimensional matrix representing the coordinates of the constraint point. The least squares fitting method is used to solve the final point set *V*‘ of the deformed region. The deformation process is shown in Fig 7(B)–7(C).

## Results

### Experimental environment

**Fig 8:**
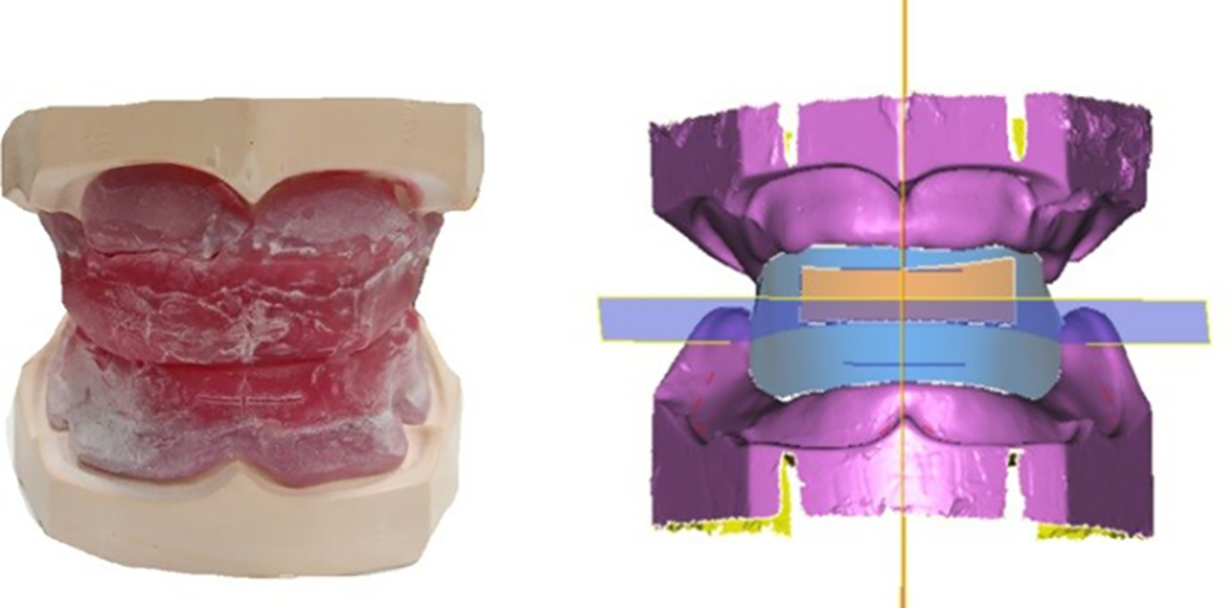
Extraction of tooth arrangement constraint information. (a) Preparation of wax occlusal rim; (b) extracted tooth arrangement constraints.

The experiment uses one edentulous plaster model and the dental base. The conventional method is used to make a wax occlusal rim, as shown in Fig 8(A). A Denmark 3Shape D700 scanner (3Shape Q-750, ± 0.02 *mm* accuracy, 3Shape, Copenhagen, Denmark) is used to scan the wax occlusal rim. The geometric constraint data that were required for the tooth arrangement, such as the occlusal plane, the fullness constraint surface and the maxillary and mandibular tooth arrangement curves, were extracted as shown in Fig 8(B). The virtual tooth arrangement calculation was developed on the VC2008 platform, and OpenGL2.0 was used for graphical display.

### Experimental results

#### Tooth arrangement results

**Fig 9:**
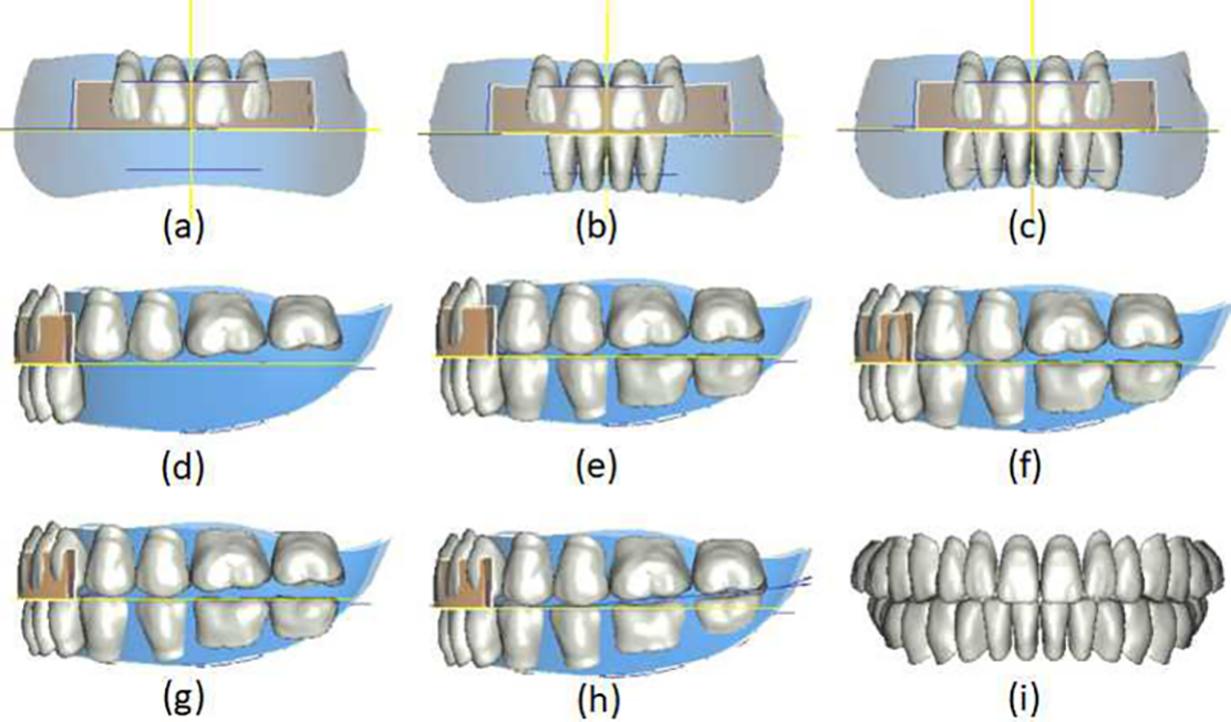
Virtual tooth arrangement. (a) Arrangement of the maxillary anterior teeth; (b) arrangement of the mandibular anterior teeth; (c) arrangement of the mandibular canines; (d) arrangement of the initial maxillary posterior teeth; (e) arrangement of the initial mandibular posterior teeth; (f) arrangement of the maxillary canines; (g) rearrangement of the maxillary posterior teeth; (h) rearrangement of the mandibular posterior teeth; and (i) front view of the full dentition.

Combining our method with the conventional tooth arrangement experience of complete denture fabrication, we developed a tooth arrangement strategy that involves the following: ① arrangement of the maxillary central and lateral incisors (Fig 9(A)); ② arrangement of the mandibular central and lateral incisors (Fig 9(B)); ③ arrangement of the mandibular canines (Fig 9(C)); ④ generation of the posterior tooth arrangement curve and arrangement of the initial maxillary posterior teeth (Fig 9(D)); ⑤ arrangement of the initial mandibular posterior teeth (Fig 9(E)); ⑥ arrangement of the maxillary canines (Fig 9(F)); ⑦ generation of the longitudinal maxillary and mandibular curves and rearrangement of the maxillary posterior teeth (Fig 9(G)); and ⑧ rearrangement of the mandibular posterior teeth (Fig 9(H)). The positions of some teeth can be fine-tuned manually. The finished dentition is shown in Fig 9(I); tooth arrangement lasted approximately 15 seconds.

#### Virtual adjusting occlusion results

**Fig 10:**
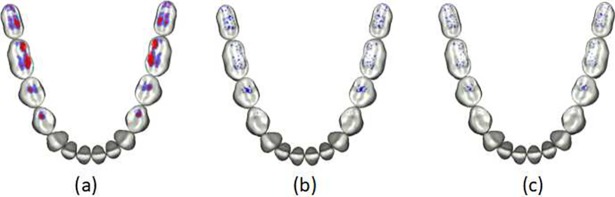
Virtual adjusting occlusion. (a) Interference region detection; (b) virtual adjusting occlusion with a maximum interference of 0.02 mm; and (c) virtual occlusion with a maximum interference of 0.01 mm.

Interference region detection of the finished dentition is shown in Fig 10(A); regions with greater than 0.05 mm interference are shown in red, and regions with less than 0.05 mm interference are shown in blue. The virtual adjusting occlusion that results when the maximum allowed interference is set to 0.02 mm and 0.01 mm are shown in Fig 10(B) and 10(C), respectively. The time required for virtual adjusting occlusion was approximately 25 seconds.

## Discussion

The present study proposed a reconfigurable rule-driven tooth arrangement method for individual edentulous patients. Four typical operators were designed and used to establish the mapping association between each characteristic standard tooth and the patient’s constraint sets. The reconfiguration of the processes using different constraint operators allowed us to flexibly achieve different clinical tooth arrangement rules. This has considerable significance for the study of automated tooth arrangement for complete dentures and for the study of tooth arrangement theory.

Most of the existing complete denture CAD software uses a three-dimensional virtual mechanical articulator to achieve occlusion. The report by Baba [13] noted that the balanced occlusion of the digital complete denture could still not satisfy the clinical requirements. Zhao et al. [18] reported that the use of a functional mandibular tracking device could achieve real-time recording of the occlusal trajectory. By combining these methods with the virtual adjusting occlusion algorithm proposed in the present paper under the support of multi-threaded technology or parallel computing and using the recorded mandibular movement trajectory of the patient, we can drive real-time biomimetic virtual adjusting occlusion. This method fundamentally overcomes the problems associated with imitation using the existing three-dimensional virtual mechanical articulator, making it possible to achieve complete denture virtual adjusting occlusion in a true sense.

In the process of tooth arrangement, when considering the constraint of the patient’s anterior or posterior tooth arrangement curve on the mesiodistal direction of a standard teeth product, it is necessary to perform scaling of the standard tooth; thus, it is difficult to directly use the commercial standard tooth in the follow-up complete denture fabrication. Besides, by the limitation of material safety, 3D printing artificial teeth cannot be popularized at present. Zhang et al. [4] used the binder jetting 3D printing process combined with a commercial dental-grade porcelain powder material to directly print the ceramic denture. This technique provides an effective means for the fabrication of individualized standard teeth.

Since conventional denture fabrication is limited by cost, quality and time, elderly patients generally use a set of complete dentures for several years. With age, alveolar bone and the mucous membranes continue to absorb, resulting in changes in the chewing system. Digital complete denture tooth arrangement technology and three-dimensional printing will improve the quality and efficiency and lower the cost of complete denture fabrication so that edentulous patients can have a pair of new complete dentures every 3 to 5 years, thereby providing better chewing function for elderly patients.

Complete denture artificial dentition and gingival red and white aesthetics are the focus of future research. The main goal of digital design of complete denture is to achieve personalized function and aesthetic restoration, the former is more important for the elderly. In the previous stage, we focused on the design algorithm of the balanced occlusion, which is closely related to the masticatory function. In the later period, our team will connect smile aesthetics design with the previous work and adjust the gingvia direction spatial relationship between the teeth and the gingiva area through the gingival margin line.

The design of complete dentures needs to meet standards of aesthetics, mechanics, production process, service life and other parameters. Thus, the design process is still complex. The integration of a digital complete denture tooth arrangement system with real-time patient mandibular trajectory recordings will further improve the accuracy of complete denture design and provide a tool for the theoretical study of a new generation of bionic complete dentures. However, there are some shortcomings of the method. The arrangement rules of artificial teeth need to be improved, such as the proportion parameters of artificial teeth need to be adjusted to obtain the better morphology. Besides, the dynamic jaw adjustment is still under study. And we cannot provide a more efficient chewing process at present.

## Conclusion

The present paper proposed a novel reconfigurable rule-driven complete denture tooth arrangement method. Combined with the virtual adjusting occlusion algorithm based on the progressive iterative Laplacian deformation, the proposed method can achieve high-precision, automated individualized tooth arrangement. When combined with a 3D printing fabrication process, the proposed method can further improve the fabrication efficiency and quality of complete dentures and lower their cost. This method might also be able to replace labor-intensive, inefficient traditional methods of complete denture fabrication technology.

## Supporting information

S1 Dataset Complete denture tooth arrangement dataset. (RAR)

S1 Video Complete denture tooth arrangement.mp4. (MP4)
